# Navigation-guided percutaneous pelvic cementoplasty for metastatic bone pain: A case report

**DOI:** 10.1097/MD.0000000000025521

**Published:** 2021-04-16

**Authors:** Ji Hyeon Lee, In Young Kim, Young Don Kim, So Young Lee, Jin Yong Jung

**Affiliations:** a Department of Anesthesiology and Pain Medicine; b Department of Neurosurgery, School of Medicine Catholic University of Daegu, Daegu, Republic of Korea.

**Keywords:** bone metastasis, navigation, percutaneous cementoplasty

## Abstract

**Rationale:**

Percutaneous cementoplasty is a minimally invasive procedure that can provide immediate pain relief and improve range of motion in patients with metastatic bone pain. Conventionally, this procedure is guided by computed tomography (CT). However, to minimize exposure to radiation, we performed percutaneous cementoplasty under the guidance of a navigation system.

**Patient concerns:**

A 60-year-old man presented with left hip pain for several months due to bone metastasis in the left ilium.

**Diagnoses:**

The patient was diagnosed with lung cancer and multiple bone metastases including ileum.

**Interventions:**

The puncture needle was placed under the guidance of a navigation system with pre-procedure CT images, and bone cement was injected into the osteolytic lesion in the left ilium.

**Outcomes:**

Bone cement placement was confirmed by post-procedure radiography, and its distribution was satisfactory. The patient's Karnofsky Performance Scale and Brief Pain Inventory scores showed improvement in pain and mobility without complications.

**Lessons:**

Percutaneous cementoplasty guided by a navigation system is a safer and more effective method with less radiation compared with conventional CT-guided methods.

## 1 Introduction

Bone metastasis is a common complication in cancer patients, especially lung cancer patients.^[1]^ Approximately 30% to 40% of lung cancer patients develop bone metastases. These metastases frequently affect the pelvic bone and cause significant pain and disability.^[2]^ Surgical treatment is an option to treat bone metastases, but in patients with extensive lytic lesions, this option could be technically difficult.^[3]^ Palliative radiotherapy could be helpful, but some patients do not respond, and bone strengthening may be delayed for patients with lytic lesions. Percutaneous cementoplasty is a minimally invasive procedure that can immediately relieve pain and restore mechanical stability.^[4]^ Conventionally, computed tomography (CT) has been used to guide percutaneous cementoplasty.^[5]^ However, cancer patients who undergo radiotherapy for primary tumors are concerned about radiation exposure associated with the procedure. Thus, navigation can be a good alternative for CT in guiding percutaneous cementoplasty.^[6]^ Here, we present a case of lytic metastasis in the left pelvis originating from the lungs. The lesion was treated by navigation-guided percutaneous cementoplasty. The application on ilium is unprecedented.

### 1.1 Ethical approval

This Paper was approved by the institutional review board of Daegu catholic university hospital, Daegu, Republic of Korea (CR-21-031). The patient has provided informed consent for publication of this case.

## 2 Case study

### 2.1 Patient characteristics

**Figure 1:**
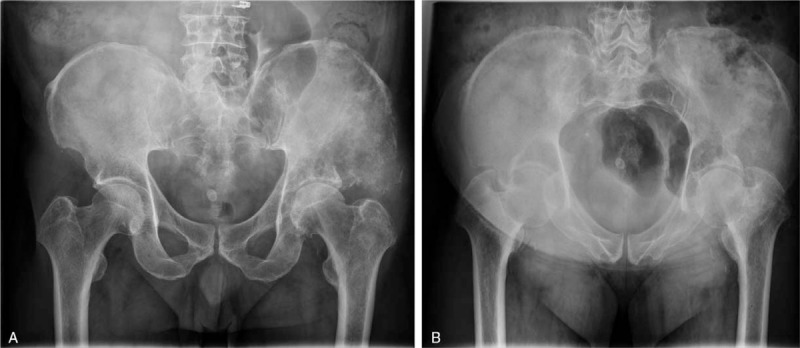
Preoperative pelvic radiographs. Anteroposterior view (A) and inlet view (B) show the osteolytic lesion in both the ilium and left acetabulum.

In April 2020, a 60-year-old man visited the orthopedic out-patient department of our institution for a chronic left hip pain. Pelvic radiography was performed, and it revealed an osteolytic lesion in both the ilium and left acetabulum (Fig. 1). In order to identify the primary cancer, enhanced chest CT was performed, which revealed a 2.4 cm solid mass in the left upper lobe apex and multiple tiny solid nodules in both lungs. However, whether the lung mass was the primary cancer was not clear. To confirm this uncertainty, bronchoscopy and percutaneous needle biopsy were performed, and the pathological findings confirmed that the lung mass was an adenocarcinoma originating from the lung.

Chemotherapy was initiated to treat the lung cancer. However, the patient persistently complained of left hip pain. Palliative radiotherapy to the left pelvic bone was performed twice without pain relief.

In October 2020, the patient was referred to a pain clinic for the persistent left hip pain. He was unable to walk or stand because of this pain. The anesthesiologist performed a femoral nerve block and targeted the muscle around the left ilium. This procedure relieved the pain for several days but not for long, and it did not improve his disability. We suggested percutaneous cementoplasty of the left ilium, which could relieve the pain by stabilizing the osteolytic lesion. Conventionally, percutaneous cementoplasty is performed under the guidance of 3-Dimensional (3-D) fluoroscopy-based CT. However, the patient was concerned about radiation exposure since he had previously been treated with radiation twice and was subjected to repetitive radiation imaging to track cancer progression. Therefore, we devised a plan to perform percutaneous cementoplasty guided by navigation in order to minimize radiation exposure.

### 2.2 Procedure technique

**Figure 2:**
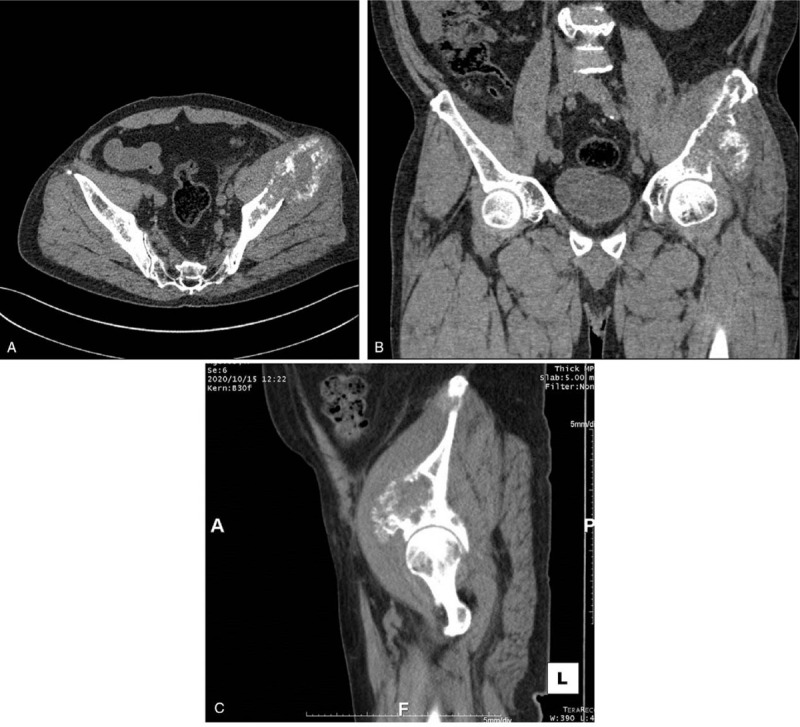
Thin-section computed tomography of the left pelvis. Horizontal view (A), coronal view (B), and sagittal view (C).

To confirm the size of the lytic lesion and perform navigation-guided cementoplasty, thin-section CT of the left pelvis was performed, with CT images sliced to 1 in. sections (Fig. 2).

Based on the pre-procedure CT images, we assumed the osteolytic lesion to be spherical with a radius of 1.3 cm. The volume of the sphere was calculated using $V=4/3\pi r^{3}=4/3\pi {\left(1.3\right)}^{3}\approx 9\;\text{mL}$. Since the lesion was not perfectly spherical and the excessive volume of the cement could cause complications such as cement leakage, we assumed the procedure could be performed safely with 6 mL of cement.

**Figure 3:**
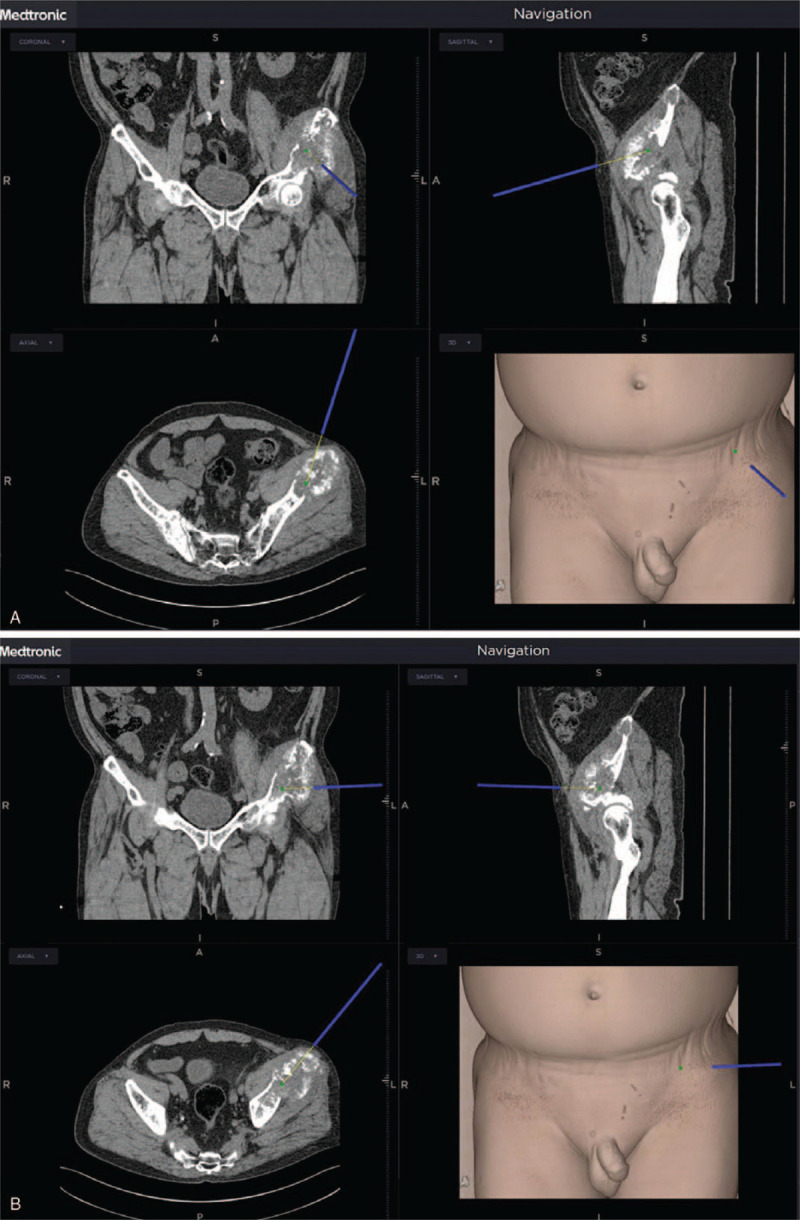
Direction and depth of the puncture needle displayed by the navigation system.

**Figure 4:**
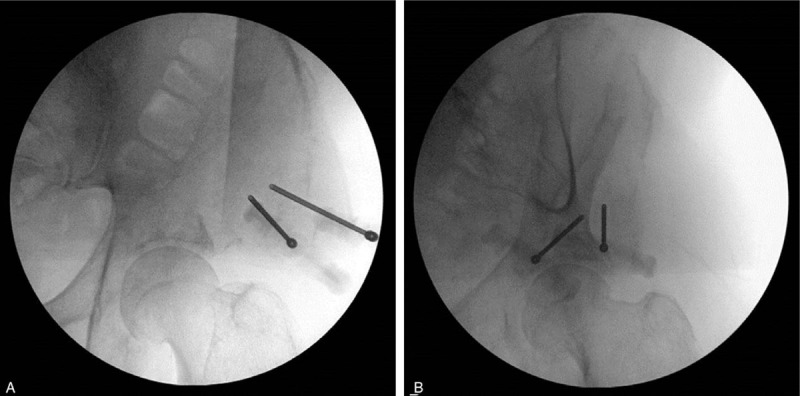
Left pelvic radiograph obtained using C-arm fluoroscopy in the operating room. Anteroposterior view (A) and oblique view (B) show the placement of the needle in the osteolytic lesion.

Thin-section CT images were uploaded to the navigation system (Medtronic Stealthstation S8, Medtronic Navigation, Inc., MN) using a USB device. The probe that was required for navigation was attached to the left flank to minimize interference with the procedure and movement. Then, the antenna of the navigation system was placed where both the treatment area and probe could be sensed. A pointer was used to touch the treatment area gently for registration, and by altering the pointer's projection depth and direction, the target point for cementoplasty could be confirmed (Fig. 3). After needle insertion, C-arm fluoroscopy was used to confirm the disposition of the puncture needle (Fig. 4). Thereafter, we injected 4 mL of cement to the medial side and 2 mL to the lateral side of the osteolytic lesion in the left pelvis.

**Figure 5:**
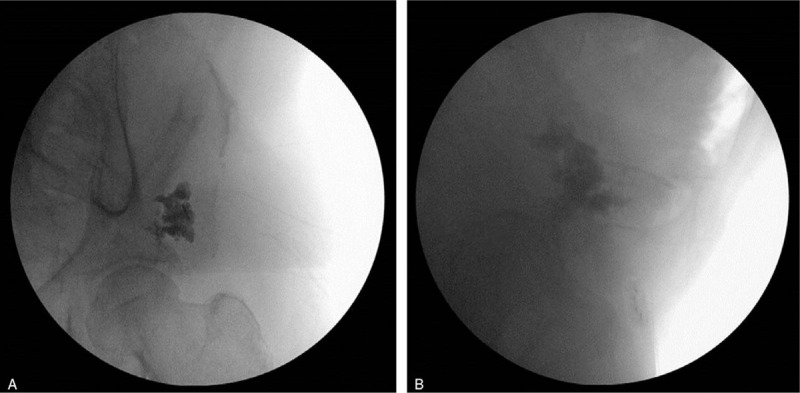
Left pelvic radiographs obtained immediately after the procedure using C-arm fluoroscopy in the operating room. Anteroposterior view (A) and oblique view (B) show the injected bone cement in left ileum.

After the procedure, C-arm radiography was used to obtain anteroposterior (AP) and oblique views of the left pelvis using in the operating room to confirm the location of the cement (Fig. 5).

**Figure 6:**
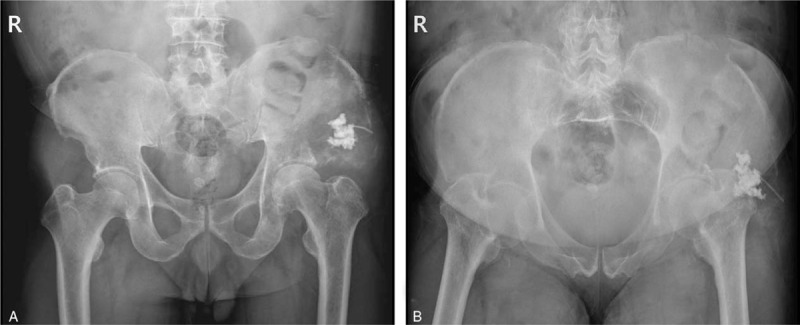
Post-procedure pelvic radiographs. Anteroposterior view (A) and oblique view (B).

Radiographs of the pelvic AP and pelvic outlet view were taken after the patient was removed from the operating room; these showed a more accurate view of the pelvis (Fig. 6).

### 2.3 Outcomes

Before the procedure, the patient's Karnofsky Performance Scale (KCP)^[7]^ score was 40 and the worst pain score on the Brief Pain Inventory (BPI)^[8]^ was 8 on a scale of 0 to 10 in the left pelvis. The general activity, mood, mechanical ability, normal work, human relations, sleep, and enjoyment of life affected by pain on the BPI were at a score of 7 on a scale of 0 to 10. To control pain, the patient was administered oxycodone 10 mg twice daily.

After the procedure, the patient's KCP score, worst pain score in BPI, and functional interference score on the BPI showed little improvement. However, on day 2, he felt dramatically better, and his KCP score improved to 60 and worst pain score on the BPI to 6. One week after the procedure, his KCP score was 70, worst pain score on the BPI was 4, and functional interference score in improved to 4 on a scale of 10. However, at 2 weeks post-procedure, the KCP and functional interference scores on the BPI returned to the pre-procedure levels because of dyspnea aggravation and newly emerged right hip pain due to right pelvic bone metastasis. Nevertheless, the worst pain score on the BPI remained constant at 4 on a scale of 10.

## 3 Discussion

Bone metastasis in the pelvis not only causes pain but also affects the mobility and other functional abilities of the patient. Traditional treatments for pain due to bone metastasis include conservative treatments such as pain medication, physiotherapy, and local anesthetic injection.^[9]^ However, these are often insufficient for metastatic cancer patients. Cementoplasty of the pelvic bone can significantly reduce pain, and by stabilizing the weakened lesion, it can improve functional movement in the treated area.^[10]^

Pain relief and increased functional ability are important targets of a treatment; however, it is also important to minimize complications because they could potentially be life-threatening or cause iatrogenic pain. Serious complications of cementoplasty include cement leakage, eccentric distribution of cement, and bone cement embolism. These complications are usually associated with disposition of the puncture needle, repeated insertion of the needle, cement viscosity, and volume of the bone cement.^[11,12]^ The navigation system is a useful tool to avoid these clinical complications because it shows the position of the puncture needle perfectly on axial, coronal, and sagittal CT images.^[6,13]^

Furthermore, the navigation guidance technique involves less radiation exposure compared with that with conventional method (3-D fluoroscopy-based CT guidance). Most patients undergoing this procedure are patients with advanced cancer and have been exposed to a lot of radiation before the procedure owing to radiotherapy and follow-up imaging for progress tracking. They choose to undergo the procedure because of severe pain and immobility. However, many patients are afraid of radiation exposure from the conventional method. Navigation-guided cementoplasty is not entirely free from radiation because a few C-arm images are acquired to confirm the needle and cement position after the procedure and prevent complications such as disposition of the needle or cement leakage. However, compared with that of 3-D fluoroscopy-based CT-guided cementoplasty, radiation exposure is greatly reduced. This is beneficial to both the patients and operator.^[6]^

In summary, percutaneous cementoplasty is an efficient method for treating metastatic bone pain by stabilizing osteolytic lesions. In addition, this procedure is a minimally invasive and could be more beneficial compared with other surgical procedures because it has a short recovery time and provides immediate pain relief with fewer complications.^[4,14]^ With a navigation system, radiation exposure can also be minimized. Since cementoplasty is usually used for terminal cancer patients as a palliative therapy, guidance methods that are less invasive and involve limited radiation exposure should be chosen. Therefore, we recommend navigation-guided percutaneous cementoplasty as the optimal treatment choice for metastatic bone pain.

## Author contributions

**Conceptualization:** So Young Lee, Jin Yong Jung.

**Data curation:** Ji Hyeon Lee, In Young Kim, Young Don Kim.

**Formal analysis:** Ji Hyeon Lee.

**Investigation:** Ji Hyeon Lee, In Young Kim, Young Don Kim, So Young Lee, Jin Yong Jung.

**Methodology:** Young Don Kim, Jin Yong Jung.

**Resources:** Jin Yong Jung.

**Software:** Young Don Kim.

**Supervision:** Jin Yong Jung.

**Validation:** Ji Hyeon Lee, In Young Kim, Jin Yong Jung.

**Visualization:** Ji Hyeon Lee, In Young Kim.

**Writing – review & editing:** Jin Yong Jung, Ji Hyeon Lee.
